# Molecular mechanisms and therapeutic advances of peritubular capillary neogenesis in acute kidney injury

**DOI:** 10.3389/fmolb.2025.1643838

**Published:** 2025-08-20

**Authors:** Yuming Ding, Linmei Gao, Yi Chen, Yanheng Qiao, Bo Yang

**Affiliations:** ^1^ Department of Nephrology, The First Teaching Hospital of Tianjin University of Traditional Chinese Medicine, Tianjin, China; ^2^ National Clinical Research Center for Chinese Medicine Acupuncture and Moxibustion, Tianjin, China; ^3^ Tianjin University of Traditional Chinese Medicine, Tianjin, China

**Keywords:** acute kidney injury, peritubular capillaries, angiogenesis, endothelial cell, pericyte

## Abstract

Acute kidney injury is a clinical syndrome characterized by a rapid decline in renal function, driven by pathological mechanisms such as renal tubular epithelial cell injury, inflammatory responses, and microcirculatory dysfunction. In recent years, the role of angiogenesis in AKI recovery and regeneration has gained increasing attention. Angiogenesis plays a dual role in tissue repair and pathological remodeling, exhibiting complex spatiotemporal dynamics during AKI progression. This review synthesizes recent advances in understanding the role of angiogenesis in AKI, with the aim of identifying potential diagnostic and therapeutic strategies. Studies indicate that the ischemic-hypoxic microenvironment following AKI activates key signaling pathways, including hypoxia-inducible factor-1α, which subsequently upregulates vascular endothelial growth factor and angiopoietins, thereby modulating intrarenal angiogenesis. Controlled angiogenesis may enhance regional perfusion, mitigate hypoxic injury, and facilitate tubular repair, whereas excessive or dysregulated angiogenesis can contribute to maladaptive vascular remodeling and fibrotic progression. Current research efforts focus on therapeutic strategies aimed at modulating angiogenesis, such as exogenous VEGF administration, endothelial progenitor cell transplantation, and Notch signaling modulation, to promote functional vascular regeneration. However, the precise role of angiogenesis varies across different AKI phases (acute vs recovery), and its interactions with inflammatory and fibrotic pathways remain incompletely understood. Further elucidation of these mechanisms is essential for developing targeted therapeutic interventions.

## 1 Introduction

Acute Kidney Injury (AKI) is a clinical syndrome defined by a rapid deterioration of renal function. This decline occurs over a short period, presenting as a reduced Glomerular Filtration Rate along with elevated serum creatinine and diminished urine output (Kellum et al., 2021). The condition constitutes a substantial global health challenge, driven by its high incidence as well as its associated mortality. Epidemiological findings show that AKI affects 10%–15% of all hospitalized patients, with this figure rising to over 50% for those in intensive care units (Hoste et al., 2018; Ostermann et al., 2025). The etiology of AKI varies markedly between different regions (Hoste et al., 2018). In high-income countries, AKI is predominantly associated with hospital-based exposures, such as major surgical procedures and nephrotoxic drugs. In contrast, infections are the primary driver of AKI in low-income regions Moreover, AKI, together with its complications, contributes to more than two million deaths globally each year. The severity and duration of the injury are critical predictors of its progression to acute kidney disease and, subsequently, chronic kidney disease (CKD). Therefore, AKI has been established as a significant risk factor for long-term renal impairment (Lameire et al., 2021). As a result, elucidating the mechanisms of post-AKI repair has become an urgent priority in nephrology research.

The renal tubules are encircled by the peritubular capillaries (PTCs), which form a dense and intricate microvascular network. These capillaries are indispensable for supplying oxygen as well as essential nutrients to tubular epithelial and interstitial cells; they also serve as critical structures for substance exchange between the tubules and the systemic circulation (Kida, 2020). During AKI, a common pathological feature is the rarefaction of these PTCs. The destruction of endothelial cells (ECs), together with physical damage to the capillaries, can culminate in their irreversible loss (Gerhardt et al., 2021). Functional recovery after an AKI episode, therefore, depends heavily on preserving PTCs endothelial integrity along with maintaining pericyte stability (Bábíčková et al., 2017).

In this setting, angiogenesis—the physiological process of forming new blood vessels from pre-existing ones—represents a vital reparative response. Through this process, the kidney attempts to restore tissue perfusion and, in turn, renal function following ischemic injury (Lerman and Chade, 2009). Taking the central role of PTCs damage in AKI pathophysiology as an instance, it is clear that these capillaries have a limited capacity for intrinsic repair. This limitation underscores the clinical significance of strategies designed to promote PTCs neogenesis, both as an endogenous repair mechanism and as a therapeutic target (Gaupp et al., 2023). This review will, therefore, summarize recent advancements in understanding PTCs neogenesis following AKI, seeking to provide a robust theoretical foundation and highlight potential clinical applications for managing this condition.

## 2 The renal tubule-vascular crosstalk: cellular interactions and molecular signaling in physiological homeostasis

**FIGURE 1:**
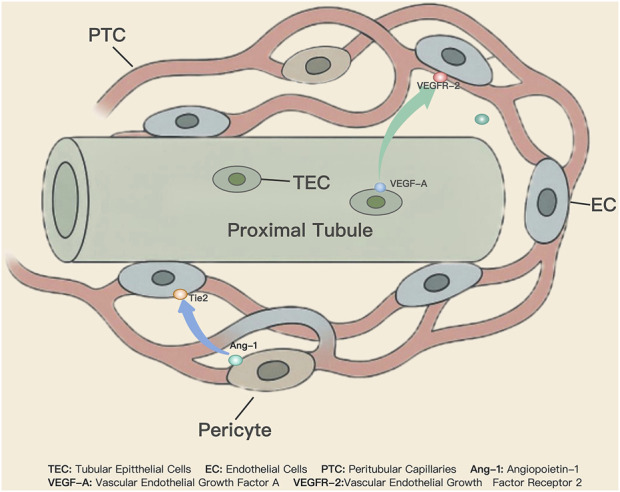
Schematic of Regulatory Mechanisms Maintaining Tubule-PTCs Homeostasis. The renal tubules and their surrounding PTCs network form the “tubulo-vascular unit,” a structure defined by close anatomical and functional integration that is essential for maintaining renal homeostasis, enabling efficient perfusion and substance exchange. Pericytes, mesenchymal cells embedded within the capillary basement membrane, play a critical role in preserving microvascular structural and functional integrity. Furthermore, the stability of ECs in the PTCs is dependent on the synergistic action of multiple signaling pathways, including the Angiopoietin/Tie2 and VEGF pathways.

The renal tubules and the surrounding PTCs network, as a highly integrated “tubulo-vascular unit”, jointly maintain renal homeostasis (Figure 1). The tight anatomical coupling and functional coordination within this unit ensure that the substantial energy demands of renal tubular reabsorption are met while simultaneously enabling efficient substance exchange and perfusion (Ferenb et al., 2016). Derived from the efferent arterioles, PTCs form a dense microvascular network that closely surrounds the proximal and distal tubules in the renal cortex. PTCs supply oxygen and nutrients to the highly metabolic tubular epithelial cells (TECs), transport reabsorbed substances back into the systemic circulation, and receive secreted metabolic waste (Kida, 2020). This efficient substance exchange, mediated by complex endocrine, paracrine, and hormonal signaling systems, is termed the renal tubule-vascular crosstalk (Ferenb et al., 2016).

Pericytes, as mesenchymal cells embedded within the capillary basement membrane, are essential for maintaining the structural and functional integrity of microvessels (Venkatachalam and Weinberg, 2017). Through direct contact with ECs and paracrine signaling, pericytes enhance the integrity of vascular junctions and reduce vascular permeability (Huang, 2020). The loss or dysfunction of pericytes causes swelling of ECs, a decreased expression of vascular homeostasis markers, and ultimately, the rarefaction of PTCs (Freitas and Attwell, 2022). Pericytes are also a primary source of vascular basement membrane synthesis. This is supported by gene expression profiling, which reveals that pericytes express multiple basement membrane matrix components, and by *in vitro* co-culture systems, which demonstrate that a structurally intact basement membrane requires the presence of pericytes for its formation (Lemos et al., 2016). Furthermore, pericytes express myosin and exhibit contractility, allowing for the fine regulation of local microcirculatory blood flow, while renal pericytes can synthesize angiotensinogen, suggesting the presence of a local renin-angiotensin system that precisely regulates PTCs blood flow (Stefanska et al., 2021).

Under physiological conditions, ECs of PTCs maintain a state of low proliferation and functional stability, a process governed by the coordinated action of multiple signaling pathways. Among these, the Angiopoietin/Tie2 and VEGF pathways represent the core regulatory axes. In healthy kidneys, pericytes release Angiopoietin-1 (Ang-1), a natural agonist of the receptor tyrosine kinase Tie2, via paracrine signaling. Ang-1 activates the Tie2 receptor on adjacent ECs, initiating downstream signaling cascades that effectively suppress inflammatory responses, reduce vascular leakage, and promote ECs survival, thus maintaining blood vessels in a mature and stable quiescent state (Chen-Li et al., 2024). Meanwhile, the VEGF pathway provides critical trophic support for endothelial cells. Vascular endothelial growth factor-A (VEGF-A), continuously expressed by podocytes in the glomeruli and TECs in healthy kidneys, acts on the VEGFR-2 receptor of ECs in PTCs (Kim and Goligorsky, 2003). This interaction is essential for maintaining the unique fenestrated phenotype of ECs and ensuring their survival. Notably, the effect of VEGF-A exhibits strictly dose-dependent; physiological levels are necessary for homeostasis, whereas dysregulated expression can induce pathological changes (Maeshima and Makino, 2010).

## 3 Pathophysiological mechanisms of AKI: from tubular injury to PTCs rarefaction

**FIGURE 2:**
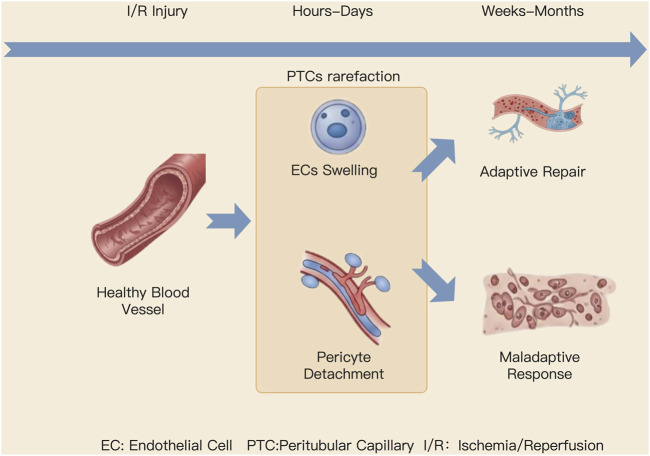
Schematic of the Dynamic Pathological Evolution of PTCs during AKI Progression. This figure illustrates the progression of AKI along a left-to-right timeline. Following an I/R insult to healthy, intact vasculature, ECs swelling and pericyte detachment occur within hours to days, culminating in PTCs rarefaction. Over the subsequent weeks or months, the course of AKI can diverge into two distinct outcomes: it may proceed toward adaptive repair, evidenced by the formation of structurally complete new blood vessels; alternatively, it can result in maladaptive repair, characterized by persistent PTCs rarefaction and extensive deposition of the extracellular matrix.

The pathogenesis of AKI is not a consequence of isolated cellular damage but is fundamentally rooted in the disruption of the “tubulo-vascular unit’s” structural and functional integrity Figure 2. Initial insults, such as ischemia, trigger concurrent pathological cascades in both the renal tubules and the microvasculature. This detrimental crosstalk between injured cells initiates a vicious cycle of “hypoxia-inflammation-vascular rarefaction-fibrosis” culminating in the progressive loss of renal structure and function (Wang et al., 2022).

### 3.1 Ischemia/reperfusion and the inflammatory cascade: a synergistic attack

Ischemia/reperfusion (I/R) injury is a common cause of AKI in clinical settings such as kidney transplantation, major surgery, and shock (Chen et al., 2025). Specific regions of the kidney, notably the S3 segment of the proximal tubule and the thick ascending limb of the loop of Henle in the outer medulla, are particularly susceptible to I/R injury due to their high metabolic activity and oxygen demand (Kwiatkowska et al., 2023). The ischemic phase induces rapid depletion of adenosine triphosphate (ATP), causing cellular energy failure, mitochondrial dysfunction, and a massive generation of reactive oxygen species (ROS). While reperfusion is essential for cell survival, it paradoxically exacerbates tissue damage through a cascade of molecular events (Nakamura et al., 2024). Damaged cells release damage-associated molecular patterns (DAMPs), such as high mobility group box-1, which, in conjunction with pathogen-associated molecular patterns in septic AKI, activate pattern recognition receptors on immune and renal parenchymal cells (Zhang et al., 2024; Tang et al., 2012). This activation triggers a massive release of pro-inflammatory cytokines (e.g., TNF-α, IL-6) and chemokines (e.g., MCP-1/CCL2), establishing a self-perpetuating, pro-inflammatory feed-forward circuit (He et al., 2025; Rabb et al., 2016). Severe energy depletion can induce necroptosis in TECs, a process mediated by the necroptosome, a complex formed by receptor-interacting protein kinase 1 (RIPK1), RIPK3, and their substrate, mixed lineage kinase domain-like protein (MLKL) (Li et al., 2024; Li et al., 2022; Gardner et al., 2023). The DAMPs released from necroptotic cells activate inflammatory responses, creating a “necrosis-inflammation” vicious cycle that accelerates kidney damage (Roger et al., 2023; Frank and Vince, 2019).

During AKI, hypoxia and inflammation are synergistic and antagonistic. Hypoxia, a consequence of microvascular dysfunction, not only directly damages cells and exacerbates endothelial dysfunction but also sensitizes the tissue to inflammatory insults (Takeda and Fong, 2007). Conversely, inflammatory mediators can inhibit mitochondrial respiration, further impair ATP production and perpetuating the cycle of injury (Gouriou et al., 2020). At the molecular level, the hypoxia-inducible factor-1α (HIF-1α)-driven hypoxia-sensing pathway exhibits significant crosstalk with the nuclear factor-κB (NF-κB)-regulated inflammatory pathway (Li et al., 2021). Inflammatory stimuli can enhance HIF-1α transcription, sustaining a state of functional hypoxia that promotes disease progression and maladaptive repair (Taylor et al., 2016). In clinical scenarios like kidney transplantation where I/R injury is unavoidable, ischemic preconditioning (IPC), namely, applying brief, non-lethal episodes of ischemia/reperfusion before a sustained ischemic event, can induce tissue tolerance to subsequent injury (Ortega-Trejo and Bobadilla, 2023). The protective mechanisms include mitigating oxidative stress, upregulating antioxidant enzymes, and inhibiting inflammatory pathways. Although results from large clinical trials have been mixed, IPC nonetheless provides a valuable conceptual framework for the prevention and treatment of AKI.

### 3.2 PTCs rarefaction: from endothelial injury to pericyte detachment

**FIGURE 3:**
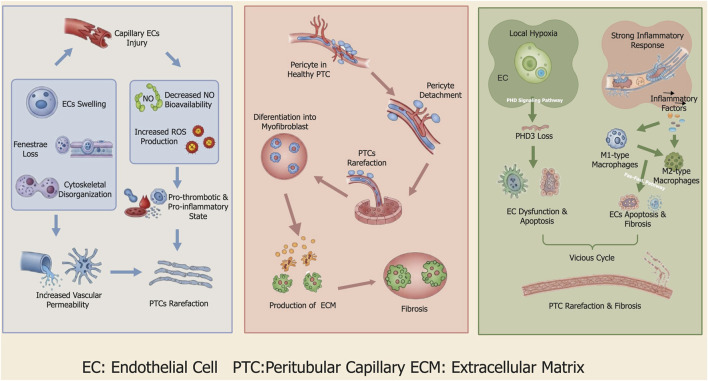
Mechanism of PTCs rarefaction. The left panel is initiated by ECs injury, which leads to morphological changes like cell swelling and fenestrae loss, increasing vascular permeability. This injury also reduces NO and elevates reactive ROS, fostering a pro-thrombotic and pro-inflammatory state that contributes to capillary loss. The left panel involves pericyte detachment, which directly destabilizes the PTCs. These detached pericytes then differentiate into myofibroblasts, producing excess ECM and driving the progression of renal fibrosis. The right panel illustrates a third pathway where local hypoxia and a strong inflammatory response drive PTC rarefaction and fibrosis. Hypoxia induces the loss of PHD3, leading to ECs dysfunction and apoptosis. Concurrently, inflammation recruits macrophages, which further promote ECs apoptosis and fibrosis. These two mechanisms create a vicious cycle that accelerates microvascular destruction, ultimately resulting in PTCs rarefaction and fibrosis.

The development of PTCs rarefaction is driven by complex cellular and molecular mechanisms, primarily encompassing ECs injury, pericyte detachment, and the synergistic effects of the local hypoxic and inflammatory microenvironment (Figure 3). The pathological process of PTCs rarefaction in AKI commences with multifaceted injury to ECs, triggered by insults such as ischemia, sepsis, or nephrotoxins (Molema et al., 2022). Morphologically, ECs injury manifests as cell swelling, loss of fenestrae, cytoskeletal disruption, and compromised vascular permeability (Finch et al., 2023). Damage to the endothelial glycocalyx, a protective proteoglycan-polysaccharide layer on the luminal surface of ECs, is a critical early event in ECs dysfunction. Inflammatory cytokines like TNF-α, in concert with enzymes such as heparanase and matrix metalloproteinases, can specifically degrade glycocalyx components, causing their shedding into the circulation (Wang et al., 2025). This process not only compromises the vascular permeability barrier but also exposes endothelial adhesion molecules, thereby promoting leukocyte adhesion and infiltration and exacerbating local ischemia (Saunders et al., 2005).

ECs injury also activates programmed cell death pathways. In septic AKI, for instance, elevated levels of the key necroptosis protein RIPK3 induce ECs membrane perforation via the RIPK1/RIPK3-MLKL pathway. Ferroptosis, characterized by decreased glutathione peroxidase 4 activity and accumulation of lipid peroxidation products, leads to ECs death through an iron-dependent lipid peroxidation mechanism (Liu et al., 2024; Jiang et al., 2025). Furthermore, ECs injury results in reduced nitric oxide bioavailability and increased ROS production, shifting the endothelial phenotype towards a pro-thrombotic and pro-inflammatory state (Yeh et al., 2024).

Pericytes are critical for maintaining the structural stability of PTCs, secreting matrix components such as type IV collagen and laminin, and regulating local blood flow through contraction. Consequently, pericyte detachment and dysfunction further aggravate microvascular injury (Venkatachalam and Weinberg, 2017). Endothelial damage disrupts the core signaling networks responsible for maintaining vascular stability. For example, dysregulation of the platelet-derived growth factor-B (PDGF-B)/PDGFRβ axis inhibits pericyte recruitment and attachment to the vessel wall (Cossutta et al., 2019). Concurrently, the excessive release of Angiopoietin-2 (Ang-2), a competitive antagonist of Ang-1, blocks Ang-1/Tie2-mediated vascular maturation signals, ultimately causing pericyte detachment from the capillary basement membrane (Park et al., 2017). Once detached, pericytes migrate into the renal interstitium and undergo a phenotypic switch upon stimulation by pro-fibrotic factors such as TGF-β (Tanaka et al., 2023). These cells then activate α-SMA expression and differentiate into myofibroblasts, which drive interstitial fibrosis through the secretion of extracellular matrix proteins like type I collagen and fibronectin (Yeh et al., 2024). Indeed, lineage tracing studies have confirmed that over 80% of α-SMA-positive myofibroblasts in fibrotic kidneys originate from the pericyte pool (Alarcon-Martinez et al., 2021). Their detachment therefore not only leads to a loss of vascular support and accelerates PTCs regression but also directly supplies the effector cells for fibrosis (Baker and Cantley, 2025).

### 3.3 The AKI-to-CKD transition: a race between regeneration and fibrosis

The progression from AKI to CKD is determined by the outcome of a “race” between adaptive repair and maladaptive fibrosis. When microvascular injury remains unresolved, the resultant persistent PTCs rarefaction creates a state of chronic tubulointerstitial hypoxia, which in turn drives the renal fibrotic process (Kida, 2020). This state of sustained hypoxia and inflammation foster a pro-fibrotic microenvironment conducive to the activation and proliferation of myofibroblasts (Kellum et al., 2021). Maladaptive repair, the core mechanism driving fibrosis, is characterized by the dysfunctional regulation of signaling pathways that possess dual roles in tissue regeneration. For example, while transient activation of the Wnt/β-catenin pathway post-injury promotes epithelial repair, its sustained or excessive activation aberrantly drives fibrotic gene expression and pathological remodeling (Bülow and Boor, 2019). The regulatory pattern of HIF-1α also demonstrates this duality: although its early, transient activation is an adaptive response to acute hypoxia, but chronic, sustained activation transforms it into a potent pro-fibrotic signal (Zhang et al., 2021). Chronic HIF-1α stabilization promotes fibrosis through multiple mechanisms, including the metabolic reprogramming of renal cells towards a pro-fibrotic phenotype and synergistic activation with pathways such as TGF-β and Notch (Liu et al., 2024).

## 4 Post-AKI renal angiogenesis: from capillary injury to repair and fibrosis

**FIGURE 4:**
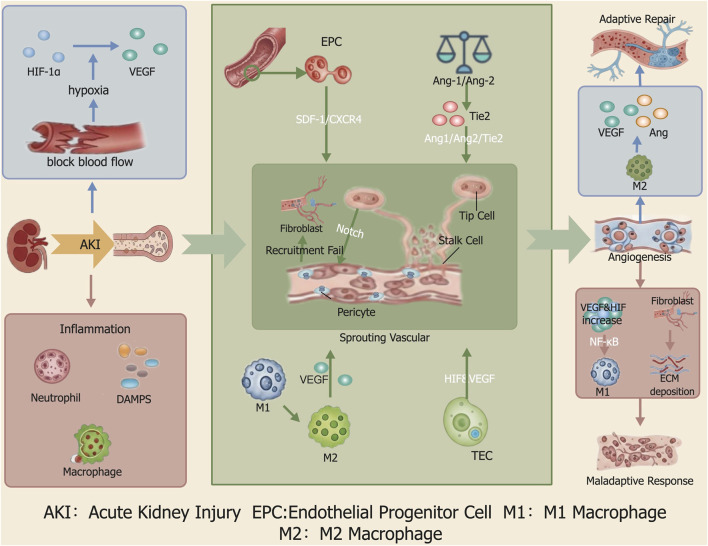
Regulatory Mechanisms of Angiogenesis Following AKI. Following AKI, hypoxia and inflammation initiate angiogenesis. This process is governed by a network of key signaling pathways: the SDF-1/CXCR4 axis mobilizes endothelial progenitor cells (EPCs); macrophage polarization from the M1 to M2 phenotype is critical for repair; the Ang/Tie2 axis controls vessel maturation; and Notch signaling ensures orderly sprouting. Successful coordination of these events promotes adaptive repair and restores renal function. Conversely, dysregulation—marked by persistent inflammation, failed pericyte recruitment, and fibroblast activation—leads to a maladaptive response, resulting in aberrant vasculature and driving renal fibrosis.

The initiation and regulation of angiogenesis in the kidney following AKI is a dynamic process involving a multitude of signaling molecules, cell types, and their complex interactions. The ultimate outcome of this angiogenic response dictates whether the kidney follows a path toward adaptive functional recovery or one toward irreversible fibrosis (Figure 4).

### 4.1 Post-injury microenvironment remodeling: the interplay of hypoxia and inflammation

As a high-perfusion organ, the kidney receives approximately 20%–25% of the cardiac output. However, even under physiological conditions, a steep oxygen gradient exists between the cortex and medulla, leaving the medulla in a state of borderline hypoxia (10–20 mmHg) due to its countercurrent exchange system (Dyson et al., 2011; Bai et al., 2022). This fragile oxygenation balance is critically dependent on the integrity of the post-glomerular microcirculation (Zhao et al., 2022). Following an AKI event, hemodynamic disturbances and increased oxygen consumption by injured and regenerating cells exacerbate this hypoxia. Hypoxia, through activation of the HIF signaling pathway, upregulates pro-angiogenic factors such as VEGF to initiate a pro-angiogenic program (Roger et al., 2023). Simultaneously, AKI triggers a robust inflammatory cascade, that leads to the infiltration of numerous immune cells, such as neutrophils and macrophages, into the damaged tissue (Yeh et al., 2024). These cells, along with injured TECs, release DAMPs, cytokines, and growth factors, forming a complex signaling network that collectively regulates angiogenesis (DeWolf et al., 2022).

Notably, the post-AKI microenvironment exerts a dual impact on angiogenesis (Tanaka and Nangaku, 2013). Early hypoxic and inflammatory signals are necessary to initiate this reparative process. However, if the inflammatory response becomes excessive or prolonged, it can damage nascent blood vessels and activate fibrotic pathways, thereby counteracting these reparative effects. Therefore, the efficacy of angiogenesis depends on the timely transition from an injurious microenvironment to a pro-reparative one.

### 4.2 The core signaling network regulating angiogenesis

HIF-1α, the master regulator of the hypoxic response, is rapidly stabilized within injured TECs and ECs (Roger et al., 2023). Activated HIFs translocate to the nucleus and, by binding to hypoxia-response elements, systematically upregulate a suite of target genes, including VEGF-A, erythropoietin (EPO), and various angiopoietins, which collectively promote ECs proliferation, migration, and new vessel formation (Thévenod et al., 2022). Furthermore, HIF activation can synergize with other signaling pathways, such as Wnt/β-catenin to promote kidney repair (Xu et al., 2022). However, the effect of HIF activation is context-dependent. While HIF signaling generally exerts a protective role in AKI by promoting angiogenesis and cell survival, its overactivation or activation in specific cell types can provoke fibrosis (Tanaka and Nangaku, 2013).

VEGF-A stimulates PTCs neogenesis by acting on VEGFR1/2 receptors on adjacent ECs; this crosstalk is mediated with greater efficiency by VEGF-A-rich extracellular vesicles secreted from TECs (Chen B. et al., 2022; Zhong et al., 2022). Renal VEGF-A expression exhibits a biphasic pattern, peaking during early AKI and again during the transition to CKD. Exogenous supplementation with VEGF-A in early AKI can be renoprotective by maintaining microvascular integrity and alleviating secondary tubular hypoxic injury. However, its dysregulation or overactivation can lead to increased permeability of nascent vessels and exacerbated inflammation (Huang et al., 2023). Moreover, different VEGF isoforms and their receptors have distinct effects: following cardiac surgery, elevated soluble VEGFR-1 increases the odds of AKI by 56%, whereas high levels of VEGF are protective. (Mansour et al., 2019).

The Ang-1/Ang-2/Tie2 signaling axis is a primary regulator of vessel stability and maturation (Liu et al., 2014). Ang-1 activates its receptor, Tie2, to promote vessel stabilization. In contrast, Ang-2, which is upregulated by ECs during AKI, functions as a context-dependent Tie2 antagonist. Initially, this antagonism facilitates vessel sprouting, but its sustained high levels disrupt vascular integrity. Vascular endothelial protein tyrosine phosphatase (VE-PTP), a key negative regulator of Tie2, is also elevated following renal ischemia (Li et al., 2023). Inhibition of VE-PTP or administration of an Ang-1 mimetic effectively mitigates AKI. Clinical data corroborate these findings, confirming that a higher Ang-1/Ang-2 ratio at hospital discharge is associated with improved long-term renal and cardiovascular outcomes (Mansour et al., 2022).

Several other pathways are also implicated in this complex process. For example, Notch signaling ensures orderly vessel growth by regulating ECs tip/stalk cell specification, but its sustained activation in TECs can induce cellular senescence and impede repair (Bakleh and Al, 2025; Sörensen-Zender et al., 2014). The SDF-1 (CXCL12)/CXCR4 axis is crucial for mobilizing endothelial progenitor cells (EPCs) to the site of injury (Cao et al., 2022). Following ischemic AKI, renal SDF-1expression increases, creating a chemotactic gradient that recruits CXCR4-positivecells to the damaged area to participate in PTCs repair.

### 4.3 Synergy and antagonism of key cellular components in vascular remodeling

PTCs remodeling is the result of concerted actions among multiple cell types. As the primary building blocks of new vessels, ECs become activated and initiate sprouting in response to angiogenic signaling cues (Almazmomi et al., 2023). The detachment of pericytes from the vessel wall is a prerequisite for initial vessel sprouting, while their subsequent re-recruitment to and ensheathment of nascent vessels is critical for vascular maturation and stabilization (Tanaka and Nangaku, 2013). However, if this pericyte recruitment fails, the detached cells can differentiate into myofibroblasts, directly driving renal fibrosis (Xu et al., 2025). EPCs are mobilized to the site of injury following AKI, where they contribute to repair either by directly integrating into the vasculature or by supporting resident ECs function through paracrine signaling (Kraińska et al., 2021). The phenotypic switch of infiltrating macrophages is a critical determinant of the repair outcome. During the repair phase, pro-inflammatory M1 macrophages must polarize toward a pro-reparative M2 phenotype for adaptive repair to proceed (Chen H. et al., 2022). These M2 macrophages actively support adaptive angiogenesis and tissue remodeling by secreting various pro-angiogenic factors and regulating the extracellular matrix.

### 4.4 The dual effects of angiogenesis: adaptive repair versus pathological transition to CKD

PTCs neogenesis during the course of AKI exhibits a tightly regulated spatio-temporal pattern that dictates its ultimate effect. Temporally, the early injury phase is characterized by PTCs structural damage; while hypoxia induces the initial release of HIF-1α and VEGF, the highly inflammatory microenvironment inhibits effective neogenesis (Baker and Cantley, 2025). The disparity between the sharp drop in ATP in proximal tubules and the more gradual decline in distal cells further confirms this injury heterogeneity (Yamamoto et al., 2020). The subsequent maintenance phase presents a divergent path: activation of repair signals can initiate mobilization of EPCs, while persistent inflammation exacerbates PTCs rarefaction (Gonsalez et al., 2019). A successful repair phase depends on the M1-to-M2 polarization of macrophages, which is required to drive ECs proliferation, lumen formation, and pericyte recruitment for vascular maturation (Chen H. et al., 2022). Furthermore, evidence suggests that the degree of ATP recovery in proximal tubules during the acute phase is negatively correlated with the extent of fibrosis in the chronic phase (Yamamoto et al., 2020). Spatially, the renal outer medulla exhibits a greater demand for neogenesis due to its high ischemic sensitivity, yet the precise spatial dynamics of this process in human AKI require further investigation (Kwiatkowska et al., 2023; Tanaka and Nangaku, 2013).

Adaptive PTCs neogenesis restores oxygen supply and facilitates the removal of metabolic waste by rebuilding the microcirculation; it also directly promotes TECs repair through paracrine factors secreted from the endothelium (Zhong et al., 2022). Clinically, elevated levels of pro-angiogenic markers such as VEGF are significantly associated with improved AKI outcomes (Mansour et al., 2019; Vakhshoori et al., 2025). Conversely, dysregulated angiogenesis can be a core driver of the AKI-to-CKD transition. Structurally immature, “leaky” new vessels can exacerbate inflammatory cell infiltration, while uncontrolled VEGF signaling can paradoxically promote M1 macrophage polarization by activating NF-κB, creating a “leakage-inflammation” vicious cycle (Tanaka and Nangaku, 2013; Ourradi et al., 2017). Persistent hypoxia and inflammation activate fibroblasts and induce excessive extracellular matrix deposition, while the aberrant, sustained activation of certain HIF and VEGF isoforms can directly promote the fibrotic process (Jiang et al., 2023). Additionally, if nascent vessels lack adequate pericyte coverage or fail to integrate into the functional circulation, their inherent defects will consume resources needed for repair, underscoring that the quality of PTCs neogenesis is as important as its quantity (Wang et al., 2023; Chou et al., 2024).

## 5 Targeting PTCs neogenesis for AKI repair: therapeutic strategies and the translational gap

### 5.1 Diagnostic and therapeutic strategies targeting PTCs neogenesis

Therapeutic strategies for AKI are undergoing a paradigm shift, moving from single-target interventions toward multi-dimensional, precise regulation of vascular repair. Growth factor-based therapies, for instance, are evolving from a simple “pro-proliferative” approach to one focused on restoring “vascular homeostasis”. Although preclinical studies show that exogenous supplementation of VEGF-A in early AKI can be renoprotective by preserving the microvasculature, its “double-edged sword” effect and the off-target risks of non-specific delivery have shifted research focus (Huang et al., 2023). Attention is now on kidney-targeted delivery and controlled-release technologies using nanocarriers, such as VEGF fused to elastin-like polypeptides. Clinical translation of these nanocarrier strategies, however, faces significant hurdles, including poor homing efficiency (Engel et al., 2019). Consequently, researchers are now focusing more on the Ang-1/Tie2 pathway, which is central to vascular stabilization (Li et al., 2023). Ang-1 mimetics, such as Vasculotide and Hepta-ANG1, have demonstrated superior protective effects and safety profiles compared to VEGF-based therapies in various animal models of AKI by restoring vascular homeostasis (Kumpers et al., 2011).

Cell-based therapies using EPCs represent another strategy. As progenitor cells capable of differentiating into mature ECs, EPCs are mobilized and home to the injured kidney following AKI, where they participate in repair through the dual mechanisms of direct integration into PTCs and paracrine signaling that improves the microenvironment (Becherucci et al., 2009; Basile et al., 2001). Notch signaling exhibits classic context-dependency in angiogenesis and kidney repair. Inhibiting Notch signaling in late-stage AKI with γ-secretase inhibitors (GSIs) can reduce fibrosis, whereas early inhibition may interfere with cell proliferation and tissue regeneration. However, the clinical use of GSIs is limited by off-target effects from systemic γ-secretase inhibition (De Chiara et al., 2021; Wyss et al., 2018). (For more details, please see the Supplementary Material).

### 5.2 Limitations in translating from animal models to the clinic

Despite extensive research revealing the mechanisms of post-AKI angiogenesis, the field faces a significant “translational gap.” Numerous therapeutic strategies that proved effective in preclinical animal models have repeatedly failed in human trials (Gerhardt et al., 2021). This disconnect stems from multiple factors. First, a therapeutic window gap exists, wherein promising preclinical strategies such as VEGF-targeted therapy and IPC have not reproduced their protective effects in clinical trials. This failure is attributed to the narrow therapeutic window created by the dual role of VEGF, the difficulty in precisely timing interventions, and the confounding effects of comorbidities and anesthetic methods on IPC within heterogeneous patient populations (Gouriou et al., 2020; Jiang et al., 2025; Bakleh and Al, 2025; H et al., 2010). Second, preclinical models suffer from a “fidelity gap.” The unilobar kidney anatomy, divergent metabolic rates and renal hemodynamics of rodents, and the absence of human-specific inflammatory pathways (e.g., involving IL-8) make it difficult for these models to fully recapitulate the immunological complexity and multi-factorial injuries seen in human AKI with comorbidities (Kida, 2020; Yamamoto et al., 2020; Gonsalez et al., 2019; Meng et al., 2023). Third, diagnostic tools, primarily serum creatinine, have a diagnostic lag. While novel biomarkers like neutrophil gelatinase-associated lipocalin can detect injury earlier, they have not yet been translated into clinically actionable guidance for therapies (Kellum et al., 2021; Wang et al., 2023; Chou et al., 2024). Similarly, while angiogenic markers like VEGF have shown prognostic value, they still lack diagnostic and therapeutic utility (Noel et al., 2024; Huang et al., 2024; Lundy et al., 2024). Collectively, these challenges constitute the translational gap for AKI angiogenesis therapies and necessitate breakthroughs in preclinical model design, the development of precise diagnostic and staging tools, and the formulation of personalized therapeutic strategies.

## 6 Future perspectives: advanced in vitro models and pro-regenerative therapies for novel target discovery

Advanced *in vitro* models, including kidney organoids and kidney-on-a-chip platforms, are revolutionizing traditional research paradigms. This transition is gaining momentum, a trend reinforced by recent policy initiatives from major regulatory bodies, such as the U.S. National Institutes of Health (NIH) and the Food and Drug Administration (FDA), to phase out animal models in favor of human-centric *in vitro* models. This transition is gaining momentum, a trend reinforced by recent policy initiatives from major regulatory bodies, such as the U.S. National Institutes of Health and the Food and Drug Administration, to phase out animal models in favor of human-centric *in vitro* models. Kidney organoids, formed through the self-organization of induced pluripotent stem cells (iPSCs) into 3D structures containing nephron-like elements, can model hereditary kidney diseases and individual drug responses when combined with patient-specific iPSCs (Kwiatkowska et al., 2023; Karp et al., 2022). However, their current lack of mature vascular networks remains a critical limitation to their application. Kidney-on-a-chip systems use microfluidics to reconstitute physiological cues like fluid shear stress and hypoxic microenvironments; for instance, the OrganoPlate® platform has successfully modeled the effects of I/R injury on renal tubules (Jiang et al., 2023; Paoli and Samitier, 2016). A current research focus is to merge the structural complexity of organoids with the functional simulation of chips to build perfusable, vascularized organoids-on-a-chip. By engineering vascular channels that anastomose with organoid microvessels, these integrated models could provide an ideal platform for studying PTCs injury and repair (Musah et al., 2024).

Building on this foundation, the integration of single-cell transcriptomics with artificial intelligence (AI)/machine learning techniques offers new avenues for precise target identification. For example, single-cell sequencing has revealed the cellular heterogeneity within injured renal tubules, identifying specific “failed-repair” cell populations that are now understood to drive fibrosis (Noel et al., 2024). AI algorithms can integrate multi-omics data with clinical information to predict AKI risk, stratify patients, and uncover novel biomarkers with greater accuracy (Rabb et al., 2016). These technologies are collectively driving the evolution of pro-regenerative therapies from single-target approaches toward spatio-temporally precise strategies (Liu et al., 2014). Furthermore, while cell-based therapies using mesenchymal stem cells show potential for repair via paracrine mechanisms, they face significant challenges related to cell survival and delivery (Lee et al., 2024). The future of AKI treatment likely lies in multi-stage, combination therapies that are “anti-inflammatory, pro-angiogenic, and anti-fibrotic,” tailored to an individual patient’s injury stage and molecular profile. Such personalized interventions will be key to finally bridging the translational gap between basic AKI research and clinical application (Lundy et al., 2024).

## 7 Conclusion

Following an episode of AKI, the injury and subsequent repair of PTCs are central events that determine the long-term prognosis of the kidney. Successful repair leads to functional recovery, whereas failed or dysregulated repair initiates a pathological progression toward chronic inflammation, fibrosis, and ultimately, irreversible kidney disease. For decades, clinical translation has stalled, largely because oversimplified preclinical models have failed to capture the heterogeneity of human AKI and because of an over-reliance on insensitive clinical biomarkers. Therapeutic development has been further complicated by the dichotomous, “double-edged sword” nature of key signaling pathways, such as the VEGF and HIF axes, which has so far precluded the precision required for their clinical application.

However, an emerging research paradigm is taking shape, centered on the convergence of advanced technologies that include *in vitro* models like kidney or ganoids, high-resolution single-cell omics, and artificial intelligence-driven analytics. This integrated approach provides the necessary tools to unravel the profound complexity of human AKI. By starting with human data, interrogating mechanisms in human-relevant systems, and identifying patient subgroups with computational precision, it is now becoming possible to overcome the limitations of past research. The ultimate goal of this new paradigm is to develop and deploy precisely timed, multi-target therapies designed to quell inflammation, promote adaptive angiogenesis, stabilize the microvasculature, and inhibit fibrosis. Such a strategy holds the promise of not only treating AKI but also preventing its devastating progression to chronic kidney disease.
