# Supplementary material for: Molecular mechanisms and therapeutic advances of peritubular capillary neogenesis in acute kidney injury
Source: Front Mol Biosci. 2025 Aug 20;12:1643838. doi: 10.3389/fmolb.2025.1643838 (PMC12404958; doi:10.3389/fmolb.2025.1643838)
Supplement: Supplementary file 1 [file Table1.docx]

Supplementary Material

# Supplementary Tables

Table 1.Therapeutic Strategies and Prognostic Biomarkers for Peritubular Capillary Injury in Acute Kidney Injury

| Strategy/Agent | Target/Mechanism | Key Findings/Evidence | Development Stage | Challenges & Limitations | Reference |
| --- | --- | --- | --- | --- | --- |
| Pro-Angiogenic Factor Therapy | | | | | |
| Exogenous recombinant VEGF-A | Activates VEGFR2, promoting endothelial cell (EC) proliferation, migration, and survival. | Early supplementation in AKI models protects microvascular structure and mitigates secondary tubular hypoxic injury. | Preclinical | Dual role: Late or excessive use may exacerbate vascular leakage and renal fibrosis. Systemic administration has off-target effects and a short half-life. | Huang et al, 2023 |
| Ang-1 mimetics (e.g., Vasculotide, Hepta-ANG1) | Activate the Tie2 receptor to stabilize blood vessels, reduce leakage, and exert anti-inflammatory effects. Counteracts ischemia-induced VE-PTP upregulation. | Effectively mitigates AKI, protects renal function, and improves endothelial integrity in murine I/R models. Aims to "stabilize" rather than "proliferate," potentially offering a safer profile. | Preclinical | Optimal timing and dosage need exploration; challenges related to the druggability of peptide/protein therapeutics. | Li et al, 2023 |
| Cell-Based Therapies | | | | | |
| Endothelial Progenitor Cell (EPC) transplantation | Homing to injury sites for direct differentiation into ECs; primarily acts via paracrine secretion of multiple pro-angiogenic and anti-inflammatory factors (e.g., VEGF, HGF, SDF-1, EVs), which can modulate pathways like the NLRP3 inflammasome. | In animal models, EPC transplantation promotes peritubular capillary (PTC) repair and improves renal function. | Preclinical | Limited cell source, difficulties in ex vivo expansion; low homing efficiency; potential for tumorigenicity and immunogenicity. | Jiang et al, 2022 |
| Signaling Pathway Modulation | | | | | |
| Notch signaling inhibitors (e.g., γ-secretase inhibitors, GSIs) | Inhibit the Notch signaling pathway (e.g., JAG1-NOTCH2 axis). | Complex role: Early inhibition may impair repair. Late-stage inhibition can mitigate renal fibrosis by preventing metabolic reprogramming (e.g., via Tfam). | Preclinical | Significant off-target effects of GSIs (e.g., gastrointestinal toxicity) necessitate the development of targeted delivery systems. | Huang et al, 2018 |
| HIF-prolyl hydroxylase inhibitors (HIF-PHIs) | Stabilize Hypoxia-Inducible Factor (HIF), upregulating target genes including VEGF and other pro-angiogenic factors. | Approved for treating renal anemia. Preclinical evidence suggests they can protect against ischemic AKI by reducing inflammation and apoptosis. | Clinical (for anemia) | Long-term, systemic HIF activation may increase risks of tumorigenesis and fibrosis; timing and safety in non-anemic AKI are unclear. | Ogawa et al, 2023 |
| SDF-1/CXCR4 axis agonists | Enhance SDF-1 signaling to promote the mobilization and homing of CXCR4+ reparative cells (e.g., EPCs) to the injured kidney. | Augmenting SDF-1 signaling can attract CXCR4+ cells to the damaged kidney, promoting repair. | Preclinical | May exacerbate inflammatory cell infiltration; systemic administration lacks specificity. | Togel & Westenfelder, 2011 |
| Diagnostic/Prognostic Biomarkers | | | | | |
| Circulating Ang-1/Ang-2 ratio | Reflects the balance between vascular stability (Ang-1) and destabilization (Ang-2). | A higher Ang-1/Ang-2 ratio at discharge in AKI patients is associated with better long-term renal and cardiovascular outcomes. | Clinical Research | Requires dynamic monitoring, as single time-point measurements have limited value; not yet a routine clinical test. | Shi & Mansour, 2023 |
| Circulating sVEGFR-1 | Acts as a decoy receptor for VEGF, inhibiting its activity. | Elevated sVEGFR-1 levels post-cardiac surgery are positively correlated with AKI risk and mortality. | Clinical Research | Levels are influenced by multiple factors, and specificity needs improvement. | Mansour et al, 2019 |

# Reference

1. Huang MJ, Ji YW, Chen JW, et al. Targeted VEGFA therapy in regulating early acute kidney injury and late fibrosis. Acta Pharmacol Sin. 2023;44(9):1815-1825. doi:10.1038/s41401-023-01070-1
2. Li Y, Liu P, Zhou Y, et al. Activation of Angiopoietin-Tie2 Signaling Protects the Kidney from Ischemic Injury by Modulation of Endothelial-Specific Pathways. J Am Soc Nephrol. 2023;34(6):969-987. doi:10.1681/ASN.0000000000000098
3. Jang HN, Kim JH, Jung MH, et al. Human Endothelial Progenitor Cells Protect the Kidney against Ischemia-Reperfusion Injury via the NLRP3 Inflammasome in Mice. Int J Mol Sci. 2022;23(3):1546. Published 2022 Jan 28. doi:10.3390/ijms23031546
4. Huang S, Park J, Qiu C, et al. Jagged1/Notch2 controls kidney fibrosis via Tfam-mediated metabolic reprogramming. PLoS Biol. 2018;16(9):e2005233. Published 2018 Sep 18. doi:10.1371/journal.pbio.2005233
5. Ogawa C, Tsuchiya K, Maeda K. Hypoxia-Inducible Factor Prolyl Hydroxylase Inhibitors and Iron Metabolism. Int J Mol Sci. 2023;24(3):3037. Published 2023 Feb 3. doi:10.3390/ijms24033037
6. Togel FE, Westenfelder C. Role of SDF-1 as a regulatory chemokine in renal regeneration after acute kidney injury. Kidney Int Suppl (2011). 2011;1(3):87-89. doi:10.1038/kisup.2011.20
7. Shi A, Mansour SG. The Role of Vascular Biomarkers in Outcomes of Patients with Kidney Disease. Nephron. 2023;147(12):778-781. doi:10.1159/000533415
8. Mansour SG, Zhang WR, Moledina DG, et al. The Association of Angiogenesis Markers With Acute Kidney Injury and Mortality After Cardiac Surgery. Am J Kidney Dis. 2019;74(1):36-46. doi:10.1053/j.ajkd.2019.01.028
